# Needle Aspiration for Severe Tracheal Compression Due to a Large Thyroid Goiter: A Case Report

**DOI:** 10.7759/cureus.80363

**Published:** 2025-03-10

**Authors:** Saketh Reddy Bathula, Nicholas Faust, Sruti Desai, Noah A Stern

**Affiliations:** 1 Otolaryngology - Head and Neck Surgery, Michigan State University, Detroit, USA; 2 Otolaryngology - Head and Neck Surgery, Detroit Medical Center, Michigan State University, Detroit, USA

**Keywords:** difficult airway management, enlarged thyroid, needle aspiration, shortness of breath (sob), tracheal compression

## Abstract

A thyroid mass is an uncommon cause of a difficult airway when planning for intubation. An enlarged thyroid may lead to difficulties with intubation and airway management, oftentimes causing tracheal compression, deviation, or both. Tracheal compression increases the risk of tracheomalacia, which could lead to possible airway collapse. Moreover, the inability to intubate on the first attempt may increase the risk of airway-related complications to the patients. Here, we present three cases of a thyroid mass with a cystic component causing tracheal deviation or compression causing shortness of breath and difficult intubation which necessitated preoperative intervention. Various techniques such as inhalational anesthetic agents, fiberoptic intubation, and awake direct laryngoscopy-aided intubation have been described in the literature but were not used due to potential complications. Needle aspiration of the thyroid cyst was performed in each case to release pressure on the trachea before intubation. No complications occurred during or following the needle aspiration procedures. Each of the three patients was subsequently intubated with a glide scope and underwent a hemithyroidectomy for definitive management.

## Introduction

An enlarged thyroid gland can lead to airway obstruction resulting in difficult endotracheal intubation [1]. According to the American Society of Anesthesiologists, a difficult airway is one in which an anesthesiologist has anticipated or unanticipated difficulty with tracheal intubation, face mask ventilation, extubation, or need for invasive airway [2]. Maguire et al. reported that about 4.9% of adult patients and 11% of infants are difficult to intubate [3]. Failure to intubate on the first attempt can increase the likelihood of complications by 33%, with complications ranging from oxygen desaturation to cardiac arrest [3]. Currently available techniques for securing airways in such patients include fiberoptic intubation (FOI), awake direct laryngoscopy-aided intubation, induction with inhaled agents, and use of preoperative angiocatheter through the cricothyroid membrane [4]. All of these airway-securing procedures may cause trauma to the airway depending on physician experience. Needle aspiration is the safest way to secure the airway in patients with severe tracheal compression caused by large cystic thyroid goiters. Here, we present three cases with severe tracheal compression due to a large thyroid mass in which successful intubation was performed following needle aspiration of the thyroid gland.

## Case presentation

Case 1

**Figure 1:**
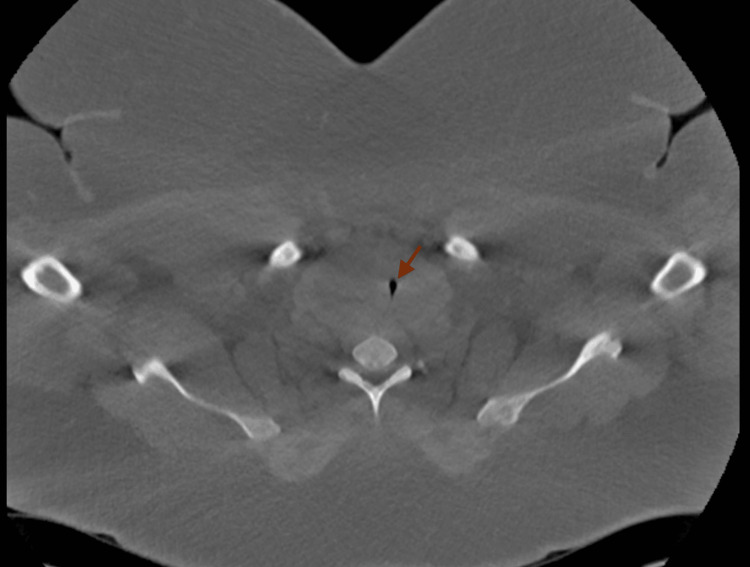
Large right thyroid mass compressing the trachea. The arrow indicates the airway compression.

A 34-year-old female with a body mass index (BMI) of 45 kg/m^2^ and past medical history of obstructive sleep apnea, hypertension, asthma, and severely enlarged thyroid, which was diagnosed six months ago, was admitted in the hospital for airway monitoring before hemithyroidectomy. She reported dyspnea and had increased work of breath, which had gradually progressed. The SpO_2_ was 93% on nasal cannula oxygen. The thyroid function tests including thyroid-stimulating hormone (TSH), T3, and free T4 were within the normal limits. Neck CT without contrast agent revealed a large right thyroid lobe cyst causing severe subglottic and cervical tracheal narrowing to 4 mm in transverse dimension (Figure 1). Thyroid ultrasound confirmed the presence of a right thyroid lobe cyst with multiple septations measuring 6.0 × 5.8 × 4.1 cm. Interventional radiology performed ultrasound-guided needle aspiration of the cyst yielding 300 cc of fluid. The patient reported immediate improvement in dyspnea. Subsequent intubation was done with a glide scope, and a number 7 endotracheal tube was inserted into the trachea. A hemithyroidectomy was performed for definitive management. No complications occurred during intubation for the procedure and the patient was extubated immediately after the surgery.

Case 2

**Figure 2:**
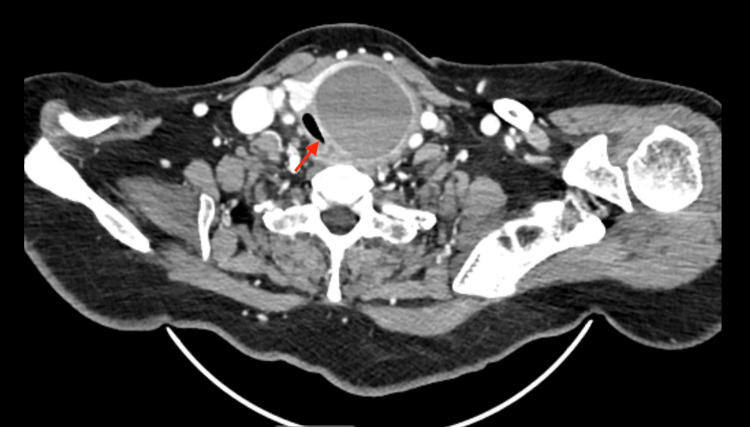
Large left thyroid mass compressing the trachea. The arrow indicates the compressed trachea.

A 64-year-old female presented to the emergency room with dyspnea, dysphagia, and noisy breathing for the past several weeks, which had worsened in the past 24 hours. She was evaluated by the emergency room physician and was suspected of having tracheal stenosis due to a large thyroid mass on the left compressing the trachea and causing shortness of breath. The thyroid function tests including TSH, T3, and free T4 were within the normal limits. The CT scan showed 3 mm of tracheal lumen at the level of the thyroid gland, implying that the patient had severe tracheal stenosis (Figure 2). Hence, the patient was deemed to have a very compromised airway and was not a candidate for emergency tracheostomy. Needle aspiration of the left thyroid cyst was performed to relieve tracheal compression. Subsequent intubation was done with a glide scope, and a number 7 endotracheal tube was inserted into the trachea for a left hemithyroidectomy. The patient was successfully extubated immediately after the thyroidectomy.

Case 3

**Figure 3:**
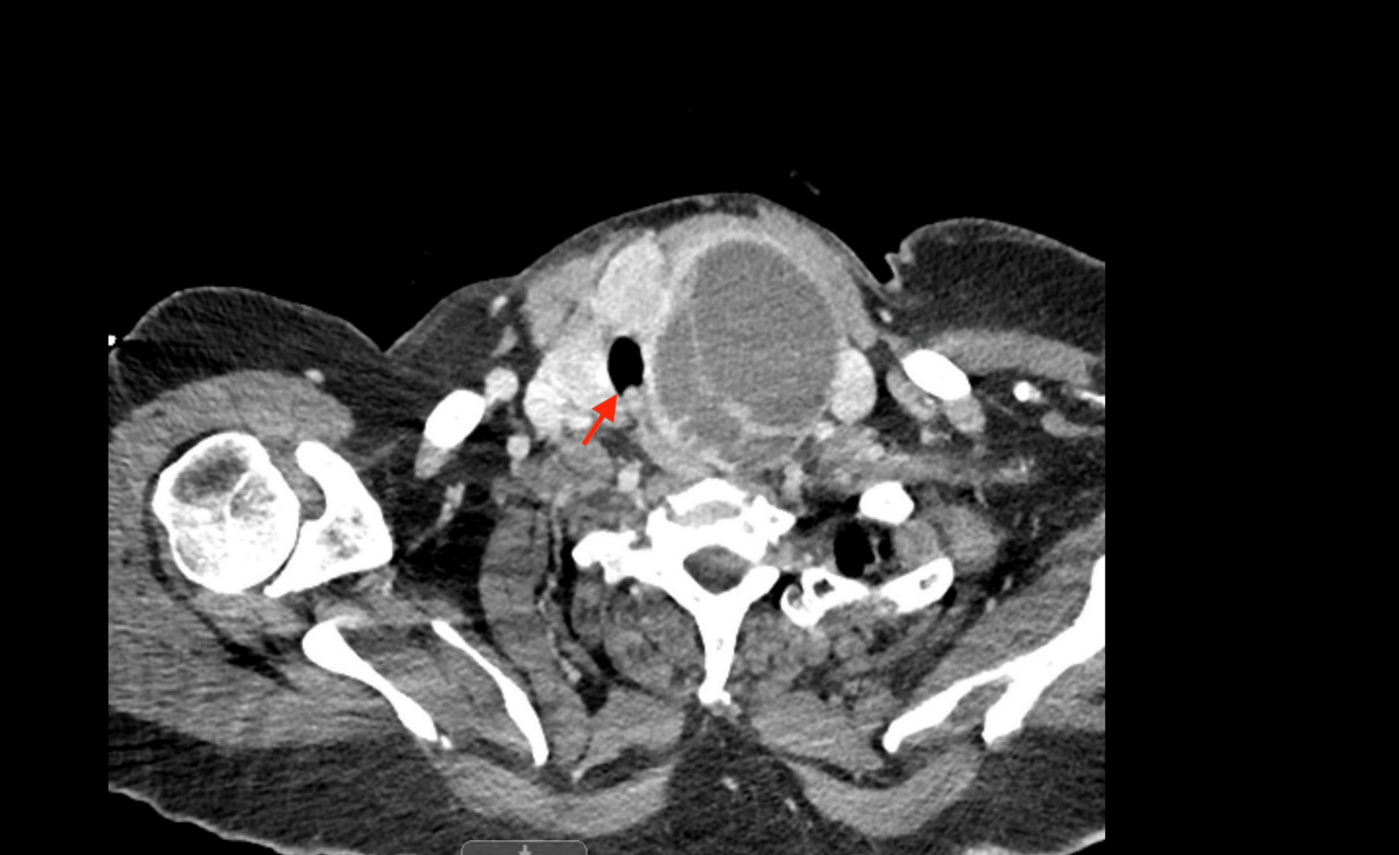
Left thyroid mass compressing the trachea. The arrow indicates the airway compression.

**Figure 4:**
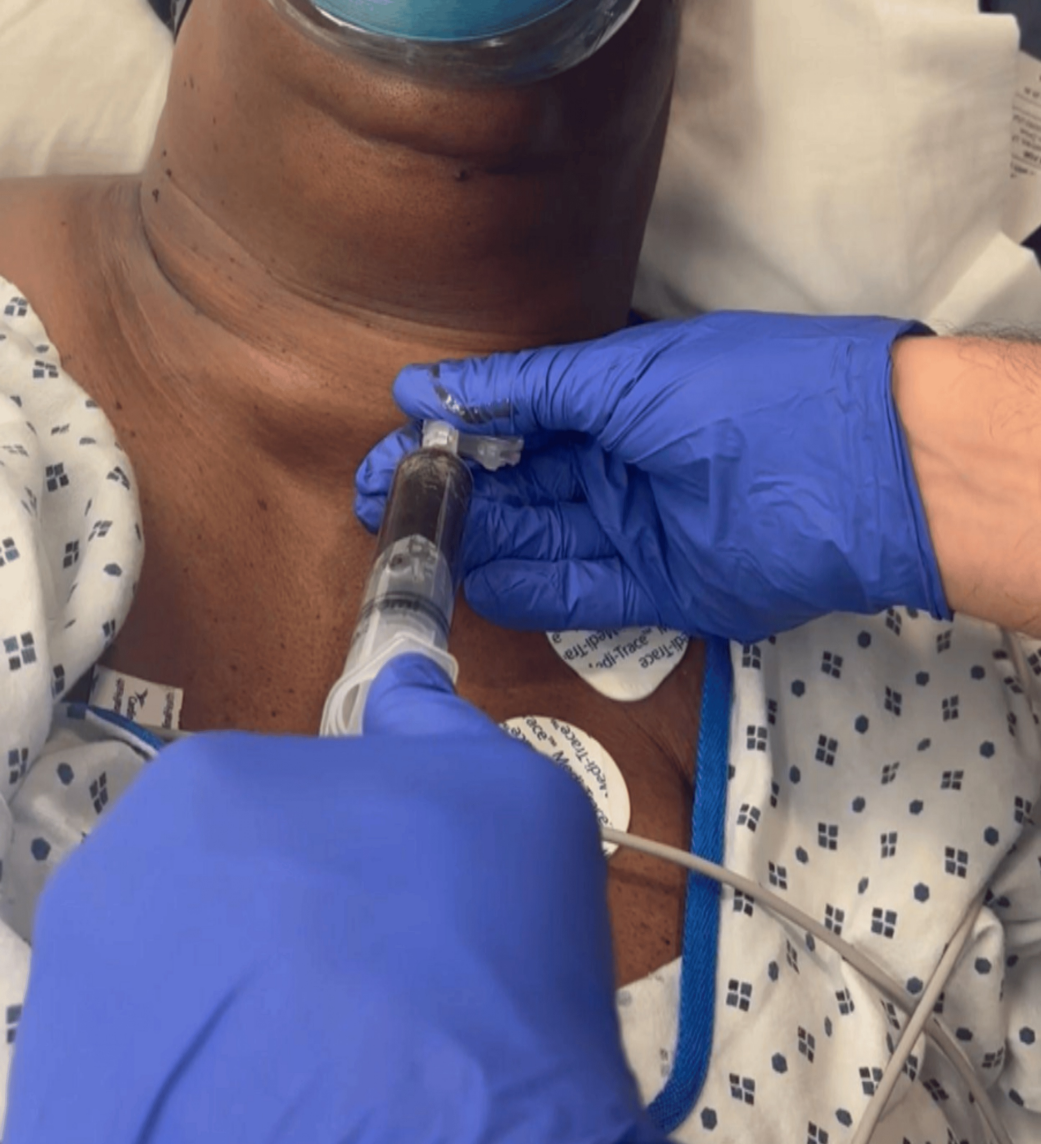
The needle aspiration procedure performed for the patient.

A 71-year-old female with no significant past medical history presented to the emergency room complaining of worsening mental status, fatigue, and lethargy for the past three or four days. She needed intubation to protect her airway due to her altered mental status and septic renal calculi removal with cystoscopy. The thyroid function tests including TSH, T3, and free T4 were within the normal limits. The ear, nose, and throat team was consulted for a large left thyroid mass with significant tracheal narrowing causing difficult endotracheal intubation (Figure 3). Needle aspiration of the left thyroid cyst was performed to relieve tracheal compression. Subsequent intubation was done with a number 7 endotracheal tube without difficulty (Figure 4).

## Discussion

Large thyroid cysts most commonly cause tracheal compression, which leads to shortness of breath [5]. According to Dean et al., ultrasonographic prevalence of thyroid cysts is 19-35% [6]. Thyroid adenoma degeneration usually leads to a fluid-filled thyroid cyst. Involution of hyperplastic thyroid follicles leads to a large follicle. Tracheal compression is noticed whenever the large thyroid follicle reaches more than 3 cm in size. Most of these large fluid-filled cysts are benign [7].

In all three cases presented, intubation was required to perform a thyroidectomy. All three patients had an enlarged thyroid cyst that was compressing the trachea and making intubation difficult. This can cause tracheal deviation, tracheal compression, or both. Induction of general anesthesia in these cases could prove risky as it may cause airway closure making intubation or mask ventilation nearly impossible [8]. Furthermore, the pressure exerted by the large thyroid cyst on the trachea can cause the tracheal wall to become soft, leading to airway collapse. Needle aspiration of the thyroid cyst is one way to release the pressure on the trachea. Other methods were not attempted due to the 3-4 mm tracheal lumen in all three patients.

FOI was not performed for these patients due to the high risk of laryngotracheal trauma, especially with a 4 mm tracheal lumen [8]. Inhalational induction using sevoflurane anesthesia can worsen the pre-existing tracheal narrowing in these patients and was not attempted. Angiocatheter through the cricothyroid membrane was also an option but was quickly disregarded as there was a large thyroid mass in these three patients which displaced the laryngeal and tracheal anatomy [9]. Awake direct laryngoscopy-aided intubation requires the full cooperation of the patient and the management of their airway reflexes, which was not possible in all three cases because the trachea was compressed with an opening of 3-4 mm [10]. Heinz et al. described how videolaryngoscopy was also not successful in previous cases of large thyroids as it does not allow for continuous ventilation, and no evidence proves that it is superior to other methodologies used in clinical situations [11]. The remaining safe option in the three cases presented was needle aspiration of the thyroid cyst. In all three cases, the fluid in the large thyroid cyst was removed through needle aspiration under local anesthesia with lidocaine without any sedation, which successfully reduced the size of the cyst. The technique reduced the pressure on the trachea allowing anesthesiologists to achieve successful intubation. A number 7 endotracheal tube is ideal to prevent tracheal wall injury and maintain better ventilation, even in obese patients. This needle aspiration procedure requires a well-trained radiologist or head and neck surgeon with ultrasound guidance. One of the disadvantages of this procedure is pain and bleeding.

## Conclusions

Needle aspiration is a viable option for patients with large thyroid cysts to relieve tracheal compression before intubation. Proper selection of patients with possible difficult airways combined with proper planning and adequate information provided to patients are indispensable to achieve the best outcomes.
